# HIV-1 Transmission linkages among persons with incident infection to inform public health surveillance

**DOI:** 10.1016/j.eclinm.2021.100968

**Published:** 2021-06-17

**Authors:** Ann M. Dennis, Simon D.W. Frost, Kimberly Enders, Andrew E. Cressman, Erik Volz, Nicole Adams, William C. Miller, Myron S. Cohen, Victoria Mobley, Erika Samoff, Joseph J. Eron

**Affiliations:** a Division of Infectious Diseases, University of North Carolina at Chapel Hill, Chapel Hill, NC, United States; b Microsoft Research, Redmond, WA, United States; c London School of Hygiene and Tropical Medicine, London, United Kingdom; d Department of Biostatistics, Gillings School of Global Public Health, University of North Carolina at Chapel Hill, Chapel Hill, NC, United States; e Imperial College, London, United Kingdom; f Division of Public Health, North Carolina Department of Health and Human Services, Raleigh, NC, United States; g Department of Epidemiology, The Ohio State University, Columbus, OH, United States

## Abstract

**Background:**

We evaluated features of HIV transmission networks involving persons diagnosed during incident HIV infection (IHI) to assess network-based opportunities to curtail onward transmission.

**Methods:**

Transmission networks were constructed using partial *pol* sequences reported to North Carolina surveillance among persons with recent (2014–2018) and past (<2014) HIV diagnoses. IHI were defined as documented acute infections or seroconversion. Demographic and virologic features of HIV genetic clusters (<1.5% pairwise genetic distance) involving ≥ 1 IHI were assessed. Persons with viral genetic links and who had diagnoses >90 days prior to an IHI were further characterized. We assessed named partner outcomes among IHI index persons using contact tracing data.

**Findings:**

Of 4,405 HIV diagnoses 2014–2018 with sequences, there were 323 (7%) IHI index persons; most were male (88%), Black (65%), young (68% <30 years), and reported sex with men (MSM) risk (79%). Index persons were more likely to be cluster members compared to non-index persons diagnosed during the same period (72% vs. 49%)*.* In total, 162 clusters were identified involving 233 IHI, 577 recent diagnoses, and 163 past diagnoses. Most IHI cases (53%) had viral linkages to ≥1 previously diagnosed person without evidence of HIV viral suppression in the year prior to the diagnosis of the IHI index. In contact tracing, only 53% IHI cases named an HIV-positive contact, resulting in 0.5 previously diagnosed persons detected per IHI investigated. When combined with viral analyses, the detection rate of viremic previously diagnosed persons increased to 1.3.

**Interpretation:**

Integrating public health with molecular epidemiology, revealed that more than half of IHI have viral links to persons with previously diagnosed unsuppressed HIV infection which was largely unrecognized by traditional contact tracing. Enhanced partner services to support engagement and retention in HIV care and improved case finding supported by rapid phylogenetic analysis are tools to substantially reduce onward HIV transmission.

**Research in context**

**Evidence before this study**

We systematically searched PubMed and Google Scholar for original research articles published between Jan 1, 2010 and Aug 1, 2020 using the following combination of search terms “HIV”, “transmission”, “network”, and “contact tracing” or “partner services”, “acute infection” or “recent infection”. Prior modeling studies in US estimated that a substantial proportion of HIV transmissions are from persons diagnosed and not receiving care. However, these studied do not include contact network data. Only three US studies with limited sample size evaluated partner services contact tracing, early HIV infection, and transmission networks.

**Added value of this study**

Our study provides an in-depth examination of documented incident (acute and very early recent) HIV infections within the context of statewide public health surveillance and standard partner services. When integrated with longitudinal viral load data, we found that over half of index persons with incident HIV infection are linked to persons previously diagnosed and without evidence of viral suppression and that case finding through partner services alone fails to identify most of these links. Our approach demonstrates utility of molecular surveillance data to (1) monitor existing interventions such as partner services, (2) identify networks with ongoing transmission where partner services can be intensified, or other network-based interventions can be employed.

**Implications of all the available evidence**

Combined with existing evidence demonstrating the burden of HIV transmission from persons diagnosed and not achieving viral suppression, our study underscores the need for improved case finding, engagement and retention in HIV care and sustained viral suppression. Future research is needed to determine how networks information that includes both socio-sexual and genetic (transmission) networks, can be leveraged to curtail sub-epidemic spread that is essential to ending the HIV epidemic in the US.

Alt-text: Unlabelled box

## 1 Introduction

HIV treatment as prevention has broad public health implications at individual and population levels, as persons receiving antiretroviral therapy (ART) who maintain viral suppression do not transmit the virus sexually [1]. But the impact of treatment as prevention is jeopardized by incomplete access and adherence to HIV care, despite overall improvements in viral suppression and ART coverage. Nearly 80% of HIV transmissions in the United States (US) appear to be from persons not diagnosed or diagnosed and not receiving care [2]. Although the estimated HIV incidence among men who have sex with men (MSM) in the US has decreased overall, substantial disparities persist by age and racial/ethnic groups. From 2010–2016, HIV incidence among MSM has remained stable among Black/African-Americans, and has increased among Latinos and among persons aged 25–35 years [3]. Similar racial and age disparities exist in achieving sustained viral suppression, posing risks for adverse health outcomes and onward transmission [4]. Southern US states, such as North Carolina (NC), face additional challenges to HIV response due to the larger burden of cases and lower rates of HIV care engagement [5].

Additional network-based tools may improve HIV case detection and prevention implementation, particularly in the Southern US [6]. Detecting and responding to acute and early infection (“incident HIV infection”), including identifying and providing partner services to the sources of ongoing transmission, will become increasingly important in achieving the goals of the federal Ending the Epidemic initiative [7]. Tracking the spatiotemporal drivers of transmission to and from high HIV burden areas is essential in “getting to zero” to adequately tailor prevention efforts towards locally driven versus imported infection. A cornerstone to public health response is Partner Services (PS), which provides linkage to HIV care and prevention services for contacts of persons with newly diagnosed HIV. Since 2002, NC has performed screening and rapid response for acute HIV infection (AHI) integrated with statewide PS [8]. But case finding is often incomplete, even during PS of incident HIV infection [9]. As people may be unable or unwilling to disclose their sexual partners or needle-sharing contacts to public health investigators. Integrating sexual network and molecular epidemiology approaches offer a detailed view of how HIV is spread within a community and could help identify opportunities to strengthen interventions using PS.

We sought to characterize the HIV genetic networks of persons diagnosed with incident HIV infection to assess probable sources of transmission and identify the demographic and virologic features of these networks that contain recent onward transmission (index persons with incident infection). Identifying clusters of genetically related infections linked to index persons could help direct intensified partner services to reach recent and past diagnoses that are disproportionately contributing to onward transmission as well as HIV-negative contacts in these networks. Sub-populations at highest risk for onward transmission could also be identified.

## 2 Methods

### 2.1 Study population

**Fig. 1:**
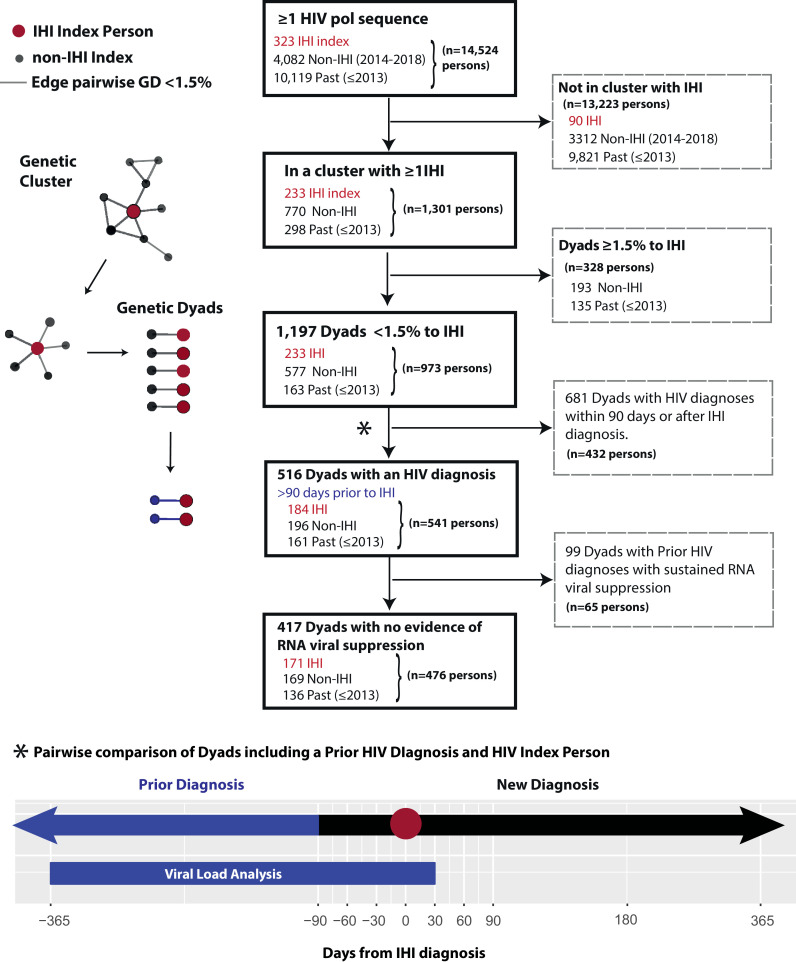
Flow diagram of study population and analyses. IHI, Incident HIV Infection; Viral suppression defined as all HIV viral load values < 200 copies/mL in a window period of 12 months prior to and within one month after the IHI index diagnosis date.

Persons ≥ 13 years old with HIV-infection residing in NC with at least one HIV *pol* sequence reported to the NC Division of Public Health (NC DPH) were evaluated. HIV partial *pol* sequences are generated by reference laboratories through clinical genotypic resistance testing and are routinely reported to NC DPH, with samples available since 2010 [10]. We assessed persons with recent versus prior HIV diagnoses; recent diagnose were persons with diagnosis dates recorded between January 1, 2014 to December 31, 2018 and prior diagnoses categorized based on diagnosis dates <2014 (Fig. 1). The study is reported in accordance with the STROBE statement for observational studies (**Supplementary Material**).

Incident HIV infection (IHI) was defined as either AHI (a negative HIV antibody test and positive HIV RNA) or early recent infection (positive HIV serology with a documented HIV negative test in the prior three months). The NC Screening and Tracing Active Transmission Program (NC-STAT) of NC DPH [8]. provides disease notification and linkage to HIV care within 72 h of the results by disease intervention specialists (DIS) for AHI and early recent infection [9].

### 2.2 nextHIV2 pipeline and cluster identification

We developed an automated cluster analysis pipeline to prospectively detect and monitor genetic clusters (nextHIV2); the pipeline is routinely updated with *pol* sequences, demographic, and laboratory data as reported to NC surveillance. Aligned sequences covering partial *pol* region (1212 base pairs; HXB2 2253–3464) are used for clustering analysis. Clusters of related sequences are generated by comparing the pairwise genetic distances (GD) with TN-93 nucleotide substitution model [11] with averaging of ambiguities. We selected pairs with <1.5% distance; all connected pairs between individuals were then linked to form clusters of connected nodes. For individuals with multiple sequences, the pair with the shortest GD was used. (see **Supplementary Material** for details methods, GD threshold selection, and complementary phylogenetic analyses).

### 2.3 Transmission risk potential and markers of HIV care receipt

Longitudinal HIV RNA viral loads (VL) from all available diagnoses were assessed. In NC, VL data are routinely reported to NC DPH with near complete reporting since 2014. Transmission risk potential was evaluated using estimates of the annual percent person time (PPT) observed >1500 copies/ml. ^4,12^ For persons with >1 VL, we calculated the annual PPT based on linear interpolation between each consecutive VL measure to estimate the number of days with >1500 copies/ml for each year of observation [12]. The date of the last available VL was assessed as a marker of care receipt in 2018. Viral suppression was defined as all VL reported in 2018 were <200 copies/mL.

### 2.4 HIV cluster and genetic dyad analyses

The HIV genetic network was constructed with all sequences with a pairwise GD < 1.5% to at least one other sequence (edges). We examined demographic and virologic features of clusters with ≥ 1 index person and assessed the contribution of past (<2014) diagnoses. We then performed a dyadic-level analysis using the dyads with ≥ 1 index person. For each dyad, we calculated the days between diagnoses between persons in the dyad. A prior diagnosis was defined as a person in the dyad who was diagnosed > 90 days prior to the index persons to account for delays in diagnosis reporting (Fig. 1). Among all probable transmission dyads that included a prior diagnosis, HIV viral suppression was estimated using VLs reported during a window period of 12 months prior or one month after the date of the index. Viral suppression was defined as all VL values in the window period < 200 copies/mL. We examined the frequency and 95% Confidence Intervals (CI) for categorical variables and the median and interquartile range (IQR) for continuous variables. Data were analyzed using R [13]. Egocentric networks were visualized using the igraph and ggraph R packages [14]. The Institutional Review Boards at the University of North Carolina and the NC DPH approved this study.

### 2.5 Comparison to partner contact tracing

We evaluated partners named and investigated for each index persons’ IHI diagnosis. The NC-DPH DIS conduct PS for sexual and needle sharing partners, focused on contacts within three months (AHI) or six months (early recent) prior to the index's diagnosis. HIV-negative partners are provided information on PrEP programs. Named partners who were previously diagnosed with detectable viral loads are referred to bridge counseling, a program where specialized DIS reach out to these individuals to assist in care re-engagement*.* For this study, we abstracted contact tracing records from the NC surveillance database for index persons and their named partners. Records are assessed for duplicates using name-based algorithms at NC DPH and were provided to the study in de-identified format.

### 2.6 Role of the funding source

The funder of the study had no role in study design, data collection, data analysis, data interpretation, or writing of the report.

## 3 Results

### 3.1 Study population

In total, 14,524 persons had a *pol* sequence reported to the NC-DPH, spanning from Oct 2010-March 2019 (*n* = 20,723 sequences). Of these, 4405 (30%) persons were diagnosed from 2014 to 2018 and 10,119 (70%) were past diagnoses (< 2014). We estimate 64% sequence coverage among diagnoses from 2014 to 2018 and 40% among adults living with HIV in NC during this period (Supplementary Material). Among recent diagnoses, 323 (7%) were index persons (incident HIV infection) (Table 1). Most IHI index persons were diagnosed during AHI (*n* = 272, 84%) and 51 (16%) were documented recent seroconverters. IHI index persons were more likely to be younger at diagnosis (median age 26 vs.31 years), report MSM risk (79% vs. 56.%) and be cluster members (72% vs. 49%) than non-IHI diagnoses [Table 1]. In total, 1301 individuals were in 162 clusters with one or more index persons, including 1003 persons diagnosed 2014–2018 and 298 (30%) past diagnoses (**Table S1**). [**Fig. S1** Distribution of Pairwise GDs] [**Fig. S2** Distribution of minimum GDs]

**Table 1:** Characteristics of persons with recent diagnoses (2014–2018) and a HIV pol sequence reported to North Carolina division of public health, stratified by diagnosis during Incident HIV infection (IHI).

**Variable**	**Not IHI Index (*n*** **=** **4082)**		**IHI Index (*n*** **=** **323)**		**Total (*n*** **=** **4405)**	
**Sex at Birth,** n% (CI)						
Male	3307	81 (80, 82)	285	88 (84, 92)	3592	82 (80, 83)
Female	775	19 (18, 20)	38	12 (9, 16)	813	19 (17, 20)
**Race/Ethnicity,** n% (CI)						
Black	2630	64 (63, 66)	209	65 (59, 70)	2839	64 (63, 70)
White	914	22 (21, 24)	78	24 (20, 29)	992	23 (21, 24)
Latino	370	9 (8, 10)	23	7 (5, 11)	393	9 (8, 10)
Other	168	4 (4, 5)	13	4 (2, 7)	181	4 (4, 5)
**Age < 30 at Diagnosis,** n% (CI)						
Yes	1892	46 (45, 48)	218	68 (62, 73)	2110	48 (46, 49)
No	2190	54 (52, 55)	105	33 (27, 38)	2295	52 (51, 54)
**Risk Group,** n% (CI)						
MSM	2285	56 (54, 58)	254	79 (74, 83)	2539	58 (56, 59)
HET-M	824	20 (19, 22)	21	065 (041, 098)	845	19 (18, 21)
HET-F	687	17 (16, 18)	35	11 (8, 15)	722	16 (15, 18)
PWID-M	159	4 (3, 5)	10	3 (2, 6)	169	4 (3, 4)
PWID-F	50	1 (1, 2)	3	<1 (<1, <1)	53	1 (<1, 2)
OTHER/UNKN	77	2 (2, 2)	0	0 (0,0)	77	2 (1, 2)
**HIV diagnosis year,** n% (CI)						
2014	770	19 (18, 20)	34	11 (7, 14)	804	18 (17, 19)
2015	861	21 (20, 22)	73	23 (18, 28)	934	21 (20, 22)
2016	961	24 (22, 25)	79	25 (20, 30)	1040	24 (22, 25)
2017	816	20 (19, 21)	79	25 (20, 30)	895	20 (19, 22)
2018	674	17 (15, 18)	58	18 (14, 23)	732	17 (16, 18)
**Field Services Unit region,** n% (CI)						
Asheville	178	4 (4, 5)	13	4 (2, 7)	191	4 (4, 5)
Charlotte	1068	26 (25, 28)	84	26 (21, 31)	1152	26 (25, 28)
Greensboro	873	21 (20, 23)	70	22 (17, 27)	943	21 (20, 23)
Raleigh	873	21 (20, 22)	102	32 (27, 37)	975	22 (21, 23)
Fayetteville	333	8 (7, 9)	16	5 (3, 8)	349	8 (7, 9)
Winterville	513	13 (12, 14)	26	8 (5, 12)	539	12 (11, 13)
Wilmington	244	6 (5, 7)	12	4 (2, 6)	256	6 (5, 7)
**HIV Subtype B,** n% (CI)						
Yes	3889	95 (95, 96)	318	99 (96, 100)	4207	96 (95, 96)
No		5 (4, 5)		2 (1, 4)		4 (4, 5)
**In Genetic Cluster,** n% (CI)						
Yes	2009	49 (48, 51)	233	72 (67, 77)	2242	51 (49, 52)
No	2073	51 (49, 52)	90	28 (23, 33)	2163	49 (48, 51)
**Partner Services,** n% (CI)						
Any contact named	—	—	259	80 (75, 84)	—	—
≥1 HIV+	—	—	170	53 (47, 58)	—	—
≥1 HIV-	—	—	137	42 (37, 48)	—	—
≥1 HIV-status unknown	—	—	95	29 (25, 35)	—	—

IHI, incident HIV infection; CI, 95% Confidence Interval; MSM, men who have sex with men; HET-F, heterosexual female; HET-M, Heterosexual male; PWID-M, male person who injects drugs; PWID-F, female person who injects drugs.

a Non-B subtypes for IHI index persons: CRF02_AG (2), CRF01_AE (2), CRF33_01B (1).

### 3.2 Contribution of people with a previous diagnosis in clusters with IHI index persons

Among 10,119 persons with HIV diagnoses before 2014, 298 (3%) were members of clusters with an IHI index. Such persons accounted for 28% of the cluster membership, yet only represented < 3% of all past diagnoses in the cohort. Persons with past diagnoses in these clusters versus not in these clusters tended to be Black (76% vs. 71%) and were significantly more likely to report MSM risk (79% vs. 38%), to be younger at diagnosis (63% vs. 30% age <26 years), spend more time viremic (estimated mean annual percent person time [PPT] of 44% vs. 37% observed with HIV RNA >1500 copies/mL from 2014 to 2018) and have no evidence of viral suppression in 2018 (42% vs. 37%) [**Table S2**.].

### 3.3 Genetic dyads involving IHI index persons

There were 1197 genetic dyads involving 973 persons (233 IHI index and 740 non-index persons [Fig. 1]. Non-index persons were predominantly diagnosed from 2014 to 2018 (78%). The characteristics between IHI index, recent non-index, and past diagnoses were similar by race/ethnicity, age at diagnosis, and MSM risk group (Table 2). Non-index recent diagnoses were more likely to be male (95% vs. 89%) and to have no documentation of viral suppression at the end of 2018 (30% vs. 22%) compared to index persons. Nearly half (48%, *n* = 357) of the non-index persons were diagnosed prior to the index in their dyad (>90 days before). Thirty (13%) index persons were in a dyad with another index with diagnosis dates >90 days apart, indicating involvement in ongoing transmission chains, directly or through shared partnerships. Most index persons in dyads (79%; *n* = 184) in were in a dyad with a prior diagnosis (median 2 dyads [IQR 1–4] with a prior diagnosis). Most prior diagnoses were diagnosed over one year before the IHI index (median 2 years IQR [1–3]) [**Fig. S3**].

**Table 2:** Characteristics of persons in genetic dyads (< 1.5% genetic distance) with an index person with an incident HIV infection (IHI), 2014–2018.

Variable	Non-index Persons				IHI index	
	Year of Diagnosis					
	<2014		2014–2018			
Total	*N* = 163		*N* = 577		*N* = 233	
**Male Sex at Birth,** n% (CI)	158	97 (93, 99)	548	95 (93, 97)	208	89 (85, 93)
**MSM Risk Group,** n% (CI)	130	80 (73, 86)	457	79 (76, 82)	189	81 (75, 86)
**Race/Ethnicity,** n% (CI)						
Black	114	70 (62, 77)	377	65 (61, 69)	150	64 (58, 71)
White	29	18 (12, 25)	129	22 (19, 26)	55	24 (18, 30)
Latino	9	6 (3, 10)	40)	7 (5, 9)	19	8 (5, 12)
Other	11	7 (3, 12)	31	5 (4, 8)	9	4 (2, 7)
**Age <30 at Diagnosis,** n% (CI)	121	74 (67, 81)	410	71 (67, 75)	160	69 (62, 75)
**Year of First Sequence,** median (IQR)	2013	(2012- 2015)	2017	(2016–2018)	2016	(2015–2017)
**Minimum% genetic distance,** median (IQR)	0.7	(0.5–1.0)	0.6	(0.3–0.8)	0.6	(0.2–0.9)
**Ever Prior Dx in IHI Dyad**^a^, n% (CI)	161	99 (96, 100)	196	34 (30, 38)	30	13 (9, 18)
**# IHI-Prior Dyads,** median (IQR)	1	(1–1)	1	(1–2)	1.0	(1–2)
**Years from Dx to IHI Dx** median (IQR)	−4	(−6.8, −2.5)	−1.0	(−2, −1)	−1	(−1, −1)
**No viral suppression in 2018**^b^, n% (CI)	80	49 (41, 57)	144	25 (21, 29)	50	21 (16, 27)
**Mean% VL >1500** median (IQR)	46	(11, 84)	17	(8- 51)	12	(5- 40)
**Mean% VL >10,000** median (IQR)	27	(5- 40)	10	(4- 31)	8	(3–27)

CI, 95% Confidence Interval; MSM, men who have sex with men; IHI, Incident HIV Infection; Dx, Diagnosis; VL, HIV RNA viral load (copies/mL).

a Prior diagnosis defined as greater 90 days prior to the diagnosis date of the index with IHI.

b Viral suppression defined as all viral load values reported in 2018 < 200 copies/mL.

### 3.4 Transmission risk potential and characteristics of prior diagnoses in genetic dyads with IHI cases

Of the 516 dyads with a prior diagnosis, 417 included a prior diagnosis without viral suppression and involved 171 (53%) of index persons (Table 3). Among these 417 dyads, most of the prior diagnoses had ≥1 VL greater than 10,000 copies/mL (50%) or no VL values reported (43%). The dyads were predominately same sex male (90%); most of prior diagnoses in the dyad were Black race (73%), resided in different counties (69%) and were similar age or younger (65%) than the index person in the dyad. Additionally, most dyads (80%) had a high degree of genetic relatedness (<1.0% GD).

**Table 3:** Characteristics of genetic dyads including index persons with incident HIV infection (IHI) and persons with prior diagnoses and no evidence of HIV viral suppression, 2014–2018.

Variable	Overall (*n* = 417)	

**Characteristic of Dyad**		
**Pairwise Genetic Distance,** n% (CI)		
< 0.5%	151	36 (32, 41)
0.5–1.0%	183	44 (39, 49)
1.0–1.5%	83	20 (16, 24)
**Sex at Birth,** n% (CI)		
Same sex Male	376	90 (87, 93)
Different sex	37	9 (6, 12)
Same sex Female	4	1 (0, 2)
**Characteristic of Prior Diagnosis in Dyad**		
**Maximum RNA viral load**^a^**,** n% (CI)		
201–1500 copies/mL	11	3 (1, 5)
1501–10,000 copies/mL	19	5 (3, 7)
> 10,000 copies/mL	208	50 (45, 55)
No viral load reported	179	43 (38, 48)
**Age at IHI diagnosis,** n% (CI)		
Same (0–5 years)	180	43 (38, 48))
Older (> 5 years)	147	35 (31, 40)
Younger (> 5 years)	90	22 (18, 26
**Current County of Residence,** n% (CI)		
Same county	118	31 (26, 36)
Different county	263	69 (64, 74)
**Years Since IHI Diagnosis,** n% (CI)		
< 1 year	149	36 (31, 41)
1–5 years	187	45 (40, 50)
5+ years	81	19 (16, 24)
**Race/Ethnicity,** n% (CI)		
Black	305	73 (69, 77)
White	61	15 (11, 18)
Other	51	12 (9, 16)
**Major Drug Resistance Mutation**, n% (CI)		
Yes	63	15 (12, 19)
No	354	85 (81, 88)

CI, 95% Confidence Interval.

a HIV RNA collected in a window period defined as 12 months prior or 1 month following the IHI diagnosis in the dyad.

### 3.5 Partners named by IHI index persons during PS contact tracing

Of the 323 index persons, 259 (80%) named at least one partner during PS (Table 1) resulting in a total of 621 unique partners investigated (**Fig. S4**). Among the 621 named partners, 25 were index persons and 596 were non-index persons at the time of the index investigation (composed of 218 HIV-positive and 378 HIV-negative/unknown status partners); 14 (3%) partners were named by more than one index. In total, 72 of the 241 named HIV-positive partners (23 index and 218 non-index partners) were new diagnoses. Two of the 25 index persons were named as partners in subsequent IHI investigations.

### 3.6 Contact tracing dyads with named HIV-Positive partners

**Fig. 2:**
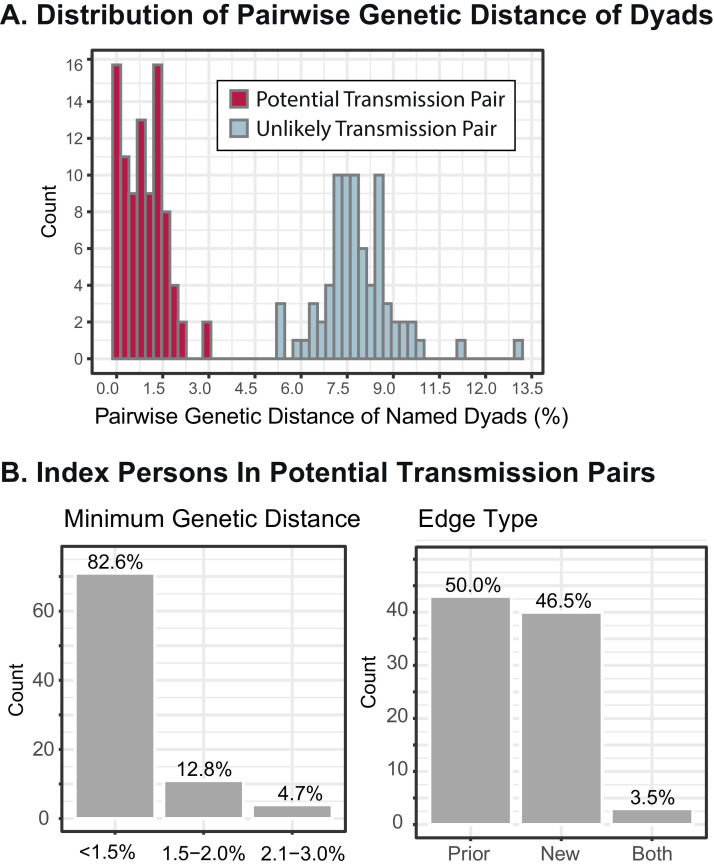
**A.** Distribution of TN-93 pairwise genetic distances among dyads with named partners and IHI persons. **B.** Distribution of minimum genetic distances and type of dyads for the 86 index persons identified in a potential transmission pair. Prior, has an edge to only a prior diagnosis; New, has an edge to only a new diagnosis; Both, had an edge to both a new and a prior diagnosis.

In total, 170 index persons (52%; 170/323) named the 241 HIV-positive partners forming 250 unique dyads (Fig. 2) resulting in 392 unique individuals. Six partners were named by more than one index, and 20 index persons were also named partners. Among these 250 HIV-positive dyads, 166 (66%) partners had sequences available and were analyzed further. Of the 166 dyads, 111 (67%) were between an index and prior diagnosis, with a median of 568 days (IQR 15–2607) between diagnosis dates. The median GD among dyads was 1.9% (IQR 0.8–7.6). Over half of dyads (54%; *n* = 90) were possible transmission pairs based on phylogenetic and pairwise GD analyses (**See Supplementary Material**); however, 16 of these dyads had GD 1.5–3.0% (Fig. 2**; Fig. S5**). In the time-scaled phylogenies of subtype B sequences, nearly all dyads with >1.5% TN-93 distance had clades dated several years prior to the index diagnosis which indicates these may not be direct transmission events (**Fig. S6).** The 166 Index-Partner dyads with sequences, involved 133 (78%) of the index persons with a named HIV-positive partner. In total, 86 (65%) of these 133 index persons were in a probable transmission pair (Fig. 2).

### 3.7 Number needed to interview to identify prior diagnoses without viral suppression

In PS, the HIV case finding yielded 0.2 (CI 0.2,0.3) new HIV diagnoses (72/323) and 0.5 (CI 0.5, 0.6) prior diagnoses (168/323) per index investigated. In other words, the number needed to interview (NNTI) was 4.5 (CI 3.7, 5.6) and 1.9 (CI 1.7, 2.2) to locate one new diagnosis and one prior HIV diagnosis, respectively. Based on the 166 dyads with sequences, we estimate an upper limit of transmission linkage of 0.65 [86/133] per index who name an HIV-positive partner. Considering that only half (53%) of index persons named an HIV-positive partner, the likelihood of PS contact tracing finding a probable transmission pair is 0.34 for any pair (0.65 × 0.53) [NNTI 2.9], 0.18 for a pair with a prior diagnosis (0.35 × 0.53) [NNTI 5.5], and 0.17 for a pair with a new diagnosis (0.32 × 0.53) [NNTI 5.8]. When considering all genetic dyads in the network at the 1.5% threshold, investigation of at least one index person can lead to identification of 1.3 (CI 1.3,1.4) [417/323] prior diagnoses without evidence of viral suppression (NNTI 0.8 [CI 0.7, 0.8]).

### 3.8 Genetic edges and DIS contact network

**Fig. 3:**
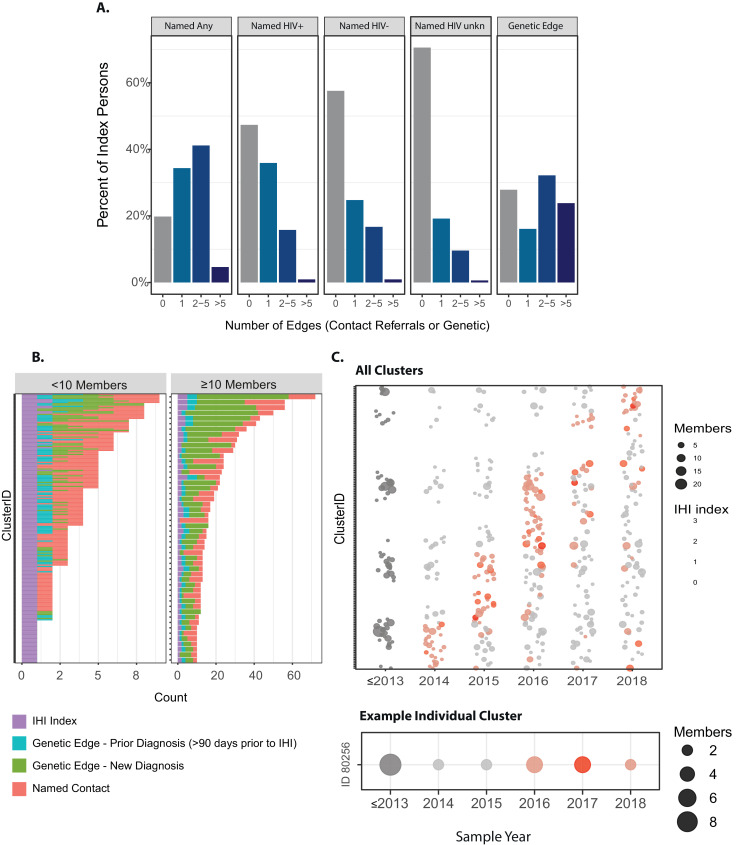
**A.** Distribution of number of named partner dyads and HIV genetic dyads among 323 index persons diagnosed with incident HIV infection (IHI) in NC, 2014–2018. **B.** Distribution of dyad type in clusters by cluster size. Persons in dyads that were prior diagnoses to the IHI index (> 90 days before) are shown in blue. **C.** Number of persons by sequence sample year for genetic clusters (For interpretation of the references to color in this figure legend, the reader is referred to the web version of this article.).

**Fig. 4:**
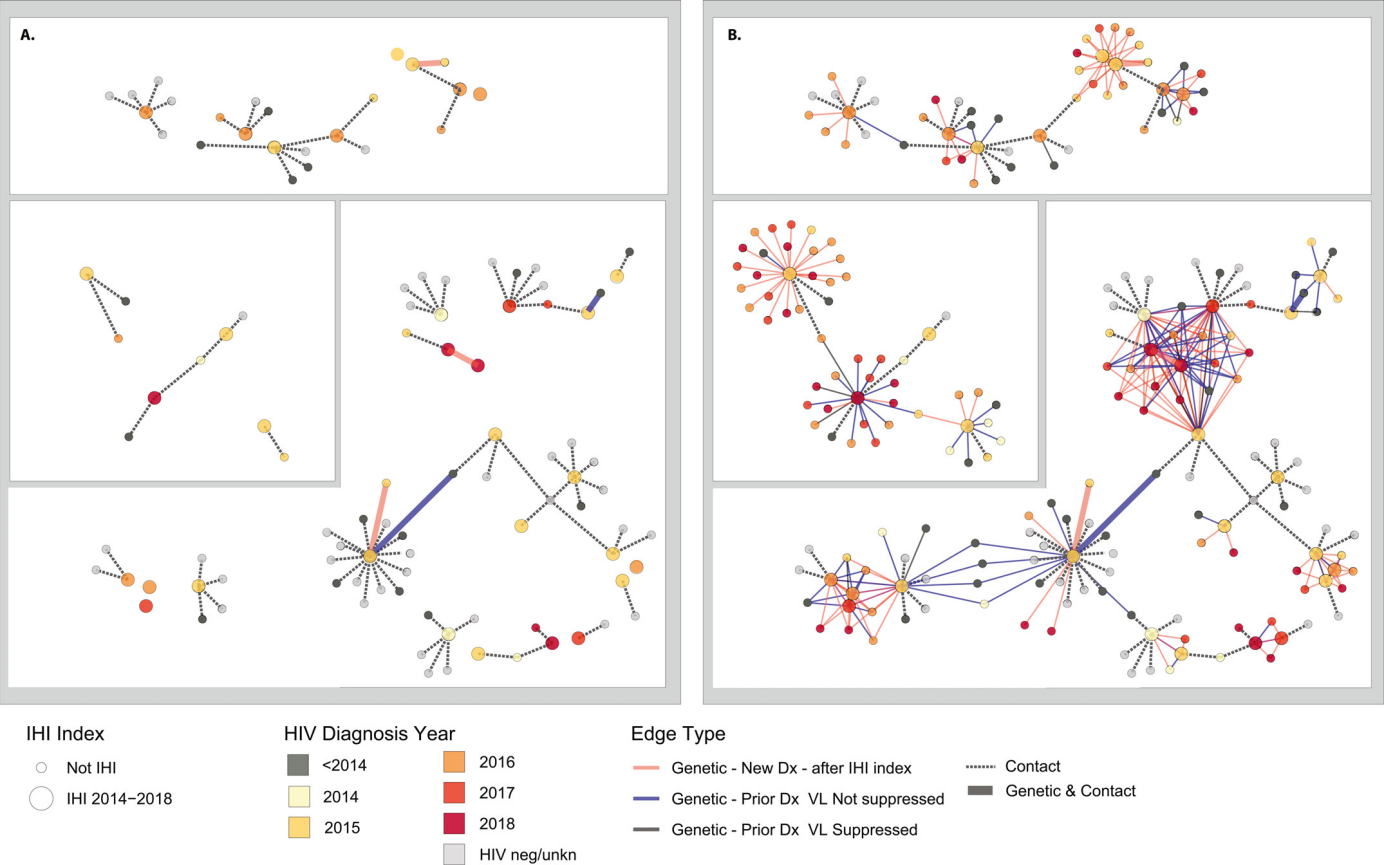
Largest three network components including HIV genetic clusters and named partner contacts among index persons diagnosed with incident HIV infection (IHI) with genetic edges (< 1.5% genetic distance) to persons with prior diagnoses (> 90 days before the IHI index) and no evidence of HIV viral suppression. Only edges < 1.5% genetic distance to IHI index are shown. **A.** Persons in these three network components who were only identified through contact tracing. **B**. All component members including those identified through HIV genetic cluster analysis.

In total, the 233 index persons in genetic dyads were distributed in 163 clusters (dyadic edges< 1.5%) [Fig. 3**B**] with a median size of 4 members (range 2–31); most clusters (70%) had only one index person. Over 75% (*n* = 122) included an edge to a new HIV diagnosis, indicating ongoing propagation of these clusters. When tracked over time (2014–2018), nearly all clusters were observed to accumulate new cases, several with multiple new IHI index persons (Fig. 3**C**). Integration of the genetic and contact network resulted in 1471 members in 173 contact network components and overlapped with 149 genetic clusters. Several of these combined contact networks span multiple genetic clusters through these partner contact referrals (**Fig. S7**). The three largest constellations that combined contact tracing and genetic clusters involved 33 index persons spanning 2014–2018 (Fig. 4). Each combined constellation includes multiple index and prior diagnoses without evidence of viral suppression in the year prior to the IHI diagnosis. With contact training alone, only 26 non-index diagnoses are identified (Fig. 4**A**). Whereas, with inclusion of the genetic dyads, 117 additional HIV-positive cases are recognized (Fig. 4**B**).

## 4 Discussion

We identified putative HIV transmission linkages for a high proportion of persons with IHI through statewide molecular surveillance. Young MSM with prior HIV diagnoses were frequently identified in genetic clusters with index persons. Over half of IHI index persons (53%) had viral genetic linkages to persons with prior HIV diagnoses (> 90 days prior) and who had no evidence of HIV viral suppression in the year prior to the incident diagnosis. These findings underscore the need for care engagement and retention interventions. These interventions could be facilitated by combined genetic and PS network-based analyses to increase identification of new HIV diagnoses and previously diagnosed person contributing to onward transmission. Network members without HIV could be prioritized for intensified PrEP and those living with HIV included in care retention programs.

Genetic clustering is increasingly used to assess HIV outbreaks, particularly among people who inject drugs [15], [16], [17] and other vulnerable groups. One of the 4 pillars of the strategic initiative to Ending the HIV Epidemic in the United States is to rapidly detect and respond to emerging clusters of HIV infection to reduce new transmissions [7]. The Centers for Disease Control and Prevention (CDC) utilizes national level data to identify and monitor rapidly growing clusters that are of priority [18] with notification to the appropriate jurisdiction. But local-level genetic cluster monitoring would enable more timely responses to local clusters due to delays in national-level data analyses and reporting [19]. NC has piloted incorporated cluster data as a prioritizing factor for partner services and linkage to testing, PrEP, and HIV care but the impact of such interventions is yet to be determined

We found that less than half of index persons with IHI referred an HIV-positive partner during contact tracing. Despite low HIV-positive case finding overall in our study, we found that 4.5 index persons need to be interviewed to yield one undiagnosed HIV case, which is more efficient than reported in other US areas [20]. Among AHI cases in San Diego, only 19% recruited a sexual partner and 15 needed to be interviewed to identify a newly diagnosed partner [20]. Assisted partner notification improved partner testing and diagnosis of HIV positive partners compared with passive referral [21]. Our study focuses predominately on AHI which is likely not representative of all recent HIV infection. Individuals diagnosed during AHI may be more likely to be in demographic groups with higher rates of HIV testing and access to healthcare. Partner recall at diagnosis during AHI may also be higher than in established infection given the recency of transmission [22]. In our study, we found that 65% of index persons with named HIV-positive partners were in potential transmission pairs. This proportion is similar to IHI in San Diego where 61% genetic linkage was reported between seroconcordant partners during tracing of early infection [20].

Over 50% of index persons were in HIV genetic clusters with persons with previous HIV diagnoses (diagnosis > 90 days before the date of the IHI), suggesting prior diagnoses remain a significant source of onward infection. In contrast, 71% of transmissions were estimated to be from recent/undiagnosed transmission among Dutch MSM [23]. Prior US studies evaluating sources of HIV transmission rely on modeling data [2,24], the largest reporting that nearly 40% of HIV transmission are estimated to be from persons who are diagnosed and not engaged in care [2]. A prior NC study estimated over 70% transmission events are attributable to previously diagnosed partners [24]. Our study is based on larger, more complete sequence sampling and leverages genetic clustering from statewide data and provides concrete evidence of the contribution of people with a prior HIV diagnosis to onward transmission acknowledging that the proportion is almost certainly an underestimation.

In the clusters with IHI index persons, we found that over half of the prior diagnoses without viral suppression had documented high HIV viremia and high estimated transmission risk potential; over 40% did not have any HIV RNA values in the prior 12 months suggesting they were not in care. A minor limitation of our study is lack of clinic visit data to confirm HIV care engagement in NC or elsewhere. Those with missing viral loads could have moved, received care outside NC or had viral loads that were not reported to NC DPH. Additionally, membership in an HIV cluster does not confirm direct viral transmission between individuals as unsampled third parties could be part of the transmission chain. Additionally, lack of observed clustering among IHI could be due to multivariant infection (which would be unrecognized with consensus pol sequences used in our analyses) or due to missingness of genotypes (only persons who have been linked to care are included as our sequence data comes from clinical samples). The latter limitation is mitigated by the high estimated statewide sampling density of sequences among newly diagnosed persons (~67%) in NC. Regardless, identifying no linkages to an IHI highlights where case finding may need to be intensified.

Our study population illustrates a particularly vulnerable group: young Black MSM, which accounted for most IHI index persons in our study and were over-represented in the previously diagnosed group who were members of clusters with IHI. Marked disparities in HIV incidence and prevalence exist among Black MSM at each step along the care continuum. Black MSM are disproportionately affected by HIV and have lower rates of engagement in HIV care and prevention; these disparities are more pronounced in the Southern US. Testing rates overall are lower among Black MSM in the South [25] and fewer Black men are consistently retained in HIV care compared to White men and Black women [26]. PrEP uptake is also under-utilized among Black MSM in the South [27]. Modeling studies suggest that disparities in transmission can be reduced but the higher prevalence will likely continue to drive higher incidence among Black MSM for decades [28].

Identifying and responding to recently transmitted HIV, the leading edge of the epidemic, is an important component of the multifaceted strategy necessary to reduce HIV transmission. However, HIV genetic cluster detection and response activities must be undertaken with community engagement and consideration of potential ethical issues [29,30]. As such public health initiatives are often conducted with waiver of participant consent, assurances to protect individual confidentiality and maintain data security are needed. Disclosing results of linkages could cause individual harm through both status disclosure as well as potential legal ramifications.

Importantly, identifying HIV transmission sources allows prioritization of resources for highest prevention impact. As HIV Field services face finite resources and large caseloads, PS and bridge counseling could be intensified for young MSM identified as cluster members to support HIV care retention and facilitate further case finding. Additionally, transmission network data could be used to prioritize HIV-negative contacts to these networks during PS. Interventions that leverage existing social network relationships and network structures can also promote behavior changes (i.e., increased HIV testing and PrEP uptake) among those at greatest risk. Altogether, such network directed interventions may have a high impact on reducing onward transmission.

## CRediT authorship contribution statement

**Ann M. Dennis:** Conceptualization, Formal analysis, Writing - Original Draft, Writing - Review & Editing, Funding acquisition. **Simon D.W. Frost:** Conceptualization Writing – review & editing. **Kimberly Enders:** Formal analysis Writing – review & editing. **Andrew E. Cressman:** Data Curation, Writing - Review & Editing. **Erik Volz:** Formal analysis, Validation, Writing - Review & Editing. **Nicole Adams:** Writing – review & editing. **William C. Miller:** Writing - Review & Editing. **Myron S. Cohen:** Writing - Review & Editing. **Victoria Mobley:** Writing – review & editing. **Erika Samoff:** Writing – review & editing. **Joseph J. Eron:** Writing - Review & Editing, Supervision.

## Declaration of Competing Interest

AMD, MSC, WCM, and JJE report grant funding by the NIH paid to their institution. SDWF reports being a paid employee of Microsoft Health Futures and receiving consulting fees through the NIH grant funding this work. MSC reports leadership roles for the HIV Prevention Trials Network, COVID-19 Prevention Network, Fogarty International Center, and McGill University International Advisory Board member. EV reports consulting fees from the University of North Carolina at Chapel Hill for scientific advice outside of this manuscript. WCM reports leadership roles with the American STD Association and as Editor-in-Chief of the STD journal and receiving honoraria for prior manuscript writing for the NIH. JJE reports clinical research contracts with Gilead Sciences, ViiV Healthcare, and Janssen paid to his institution, consulting fees paid to him by Merck, Janssen, Gilead Sciences, and ViV Healthcare, and participation on a Data Safety and Monitoring Board for Westat Inc. All these relationships occurred outside of this manuscript. AEC, ES, NA, VM, and KE report no competing interests.

## Appendix Supplementary materials

Image, application 1
