# Supplementary material for: HIV-1 Transmission linkages among persons with incident infection to inform public health surveillance
Source: eClinicalMedicine. 2021 Jun 17;37:100968. doi: 10.1016/j.eclinm.2021.100968 (PMC8225702; doi:10.1016/j.eclinm.2021.100968)
Supplement: Supplementary file 1 [file mmc1.docx]

**SUPPLEMENTARY MATERIAL**

1. **HIV *Pol* Sequence Sampling Proportion**

**Estimated proportion of HIV-positive persons diagnosed in North Carolina from 2014-2018 that are represented in the cohort of recent diagnoses (n=4,405 persons).**  In total, 6,543 persons were diagnosed with HIV infection in North Carolina from Jan 1, 2014 to Dec. 31, 2018. Among these 4,138 were in our dataset of 4,405 persons residing in North Carolina with a diagnosis reported in the same time period and with an HIV pol sequence available (267 were diagnosed outside North Carolina). Therefore, we estimate our sample represents 63% (4,138/6543) persons diagnosed with HIV infection in North Carolina from 2014-2018.

Persons with a *pol* sequence were similar to the population of NC diagnoses (2014-2018) by several characteristics (**Table S1**), however persons with sequences were more likely to be men and report MSM risk. Sequence sampling did vary by calendar year of diagnosis. From 2014-2018, the proportion of persons diagnosed and who had a sequence was 55%, 64%, 71%, 66%, and 59%, each calendar year from 2014-2018, respectively.

**Estimated Proportion of HIV-Positive Persons Living in North Carolina represented in Overall Dataset** (n=14,524 persons). As of December 31, 2018, there were 35,457 persons estimated to be living with HIV in North Carolina^1^. We estimate that the overall dataset represents approximately 41% of persons living with HIV in the state.

1. **Detailed Methods for the nextHIV2 Platform**

nextHIV2 is an automated cluster analysis pipeline to prospectively detect and monitor genetic clusters. The pipeline is routinely updated with *pol* sequences, demographic, and laboratory data as reported to NC surveillance, which are imported from a REDCap database. Deposited sequences are codon aligned against a reference sequence (HXB2) using the program bealign (http://github.com/veg/bioext), assuming an HIV-BETWEEN-F amino acid substitution matrix. Aligned sequences covering partial *pol* region (1212 base pairs; HXB2 position 2253-3464) are used for clustering analysis. Clusters of related sequences are generated by comparing the pairwise genetic distances (GD) with TN-93 nucleotide substitution model^2^ with averaging of ambiguities, as implemented in the tn93 program (<http://github.com/veg/tn93>).

**Subtypes and Circulating Recombinant Form Identification**

Subtypes were identified using the V3 Rega Subtyping Tool.^3^ Unassigned sequences were examined further in HIV COMET.^4^ We examined the pairwise GD (sub/site) stratified by major subtype/CRF (n=14,524 persons, one sequence per individual). The dominant four subtypes/CRFs among persons with new diagnoses 2014-2018 are shown in **Figure S1A and S1B**. Given the large size of the dataset, pairwise distances for subtype B are based on a random sample of 1x10^6^ observations (**Figure S1A)**.

The median Pairwise GDs for Cluster Members versus Non-Cluster Members is shown in **Figure S1C**

**Surveillance Drug Resistance Mutations**

We identified mutations by antiretroviral drug class (non-nucleoside reverse transcriptase inhibitor, nucleoside reverse transcriptase inhibitor [NRTI], protease inhibitor [PI]) using the Stanford University HIV Drug Resistance Database genotypic resistance interpretation algorithm. Major mutations were selected using the 2009 standardized list of SDRMs from the World Health Organization.^5^

**Genetic Distance Thresholds**

We selected all pairs with GD <1.5%; all connected pairs between individuals were then linked to form clusters of connected nodes. For individuals with multiple sequences, the pair with the shortest GD was used. While there is no consensus on thresholds to define HIV genetic clusters,^6^ the TN-93 clustering has previously shown efficacy for identification of transmission pairs.^7,8^ The 1.5% GD threshold was initially selected for the overall dataset as the 1.5%  threshold translates to detection of networks with a maximum of  7–8 years of viral evolution^9^ and has been widely used in molecular epidemiology studies. Because we were interested in transmission linkages with early incident HIV infection, we expected little divergence over time. Among individuals with primary HIV infection less than 1% divergence between baseline and longitudinal sequences collected up to 9 years has been reported.^10^ Several studies have shown that higher GD than 1.5% can be seen between transmission pairs using *pol* consensus sequences.^11,12^ Such divergence could occur when sequences are sampled years following the transmission event.

Given this, we further evaluated the distribution of pairwise GD to further determine suitability for the 1.5% threshold. Among the 4,405 persons with recent diagnoses, we examined the minimum GD to any other sequence in the overall dataset (n=14,524 sequences). The histogram and non-parametric estimation show a bimodal distribution **(Figure S2A)**. Using a finite mixture model with the Expectation-Maximum (E-M) algorithm,^13^ we estimated a cutoff that separates the two peaks **(Figure S2B).** We similarly examined the distribution of minimum pairwise GD for the 323 index persons with IHI (**Figure S2C and S2D**).

1. **Genetic Distance and Phylogenetic Analyses for Named HIV-Positive Partner Dyads**

We considered the distribution of pairwise GD between index persons and named partners (named dyads) [**Figure S2**]. The distribution was bimodal with median pairwise GD 0.019 (IQR 0.008-0.076) substitutions/site. However, the two modes of the distribution are quite different. The first mode, the median GD is 0.005 (IQR 0.001-0.017), the GD range 0-0.031, while the second mode has a median GD of 0.067 (IQR 0.013, 0.079). The 1.5% GD threshold in our dataset would identify 82% (74/90) pairs in the first mode (**Figure S2A**).

We evaluated phylogenetic clades membership between these dyads in maximum likelihood (ML) phylogenetic trees. We designated a dyad as a potential transmission pair if the TN-93 pairwise GD <0.031 and the dyad was part of the same phylogenetic clade with high branch support. A ML phylogenetic tree was constructed with 14,524 sequences in FastTree 2.2.10^14^ using a GTR substitution model and branch support assessed with Shimodaira-Hasegawa [SH-like] test. Clades containing sequences from the index-partner dyads were examined. In comparison with the phylogenetic tree, all dyads in the first mode are in the same clade with high branch support (>80% SH-Like test in FastTree), whereas all the dyads in the second group are not in the same phylogenetic clade. These dyads were more likely to include a prior diagnosis compared to the dyads in the first mode of the distribution (84.4% versus 51.7%) and 20% of partners in these dyads had documented viral suppression in the 12 months prior to the index diagnosis.

**Time-Scaled Tree of Subtype B Sequences from Index Persons and Named Partners**

Accurate reconstruction of dated trees requires sufficient variation in sampling dates to estimate the evolutionary rate and can be misled by including divergent and/or recombinant sequences. To estimate a dated tree for index cases (n=323) and their named partners (an additional n=148), we combined these sequences with historical sequences from North Carolina that were previously submitted to GenBank (n=1794, with sample dates from 1997-05-01 to 2014-06-26 [Accession # KY579388-KY579750, JX160108-JX161480]). Subtypes were identified using the V3 Rega Subtyping Tool^3^, and analysis was restricted to HIV-1 Subtype B onl­y (417/471 for the index and partners, 1433/1794 for the background sequences). Sequences were aligned against HXB2, and a maximum likelihood (ML) phylogeny reconstructed using IQTREE v.2.0.6^15^, assuming a GTR+G4 model of substitution, with more thorough tree search using nearest neighbor interchange (--allnni). The ML phylogeny was rooted using root-to-tip regression using the ape library in R^16^, which demonstrated sufficient temporal signal in the data. Dated trees were generated from this ML phylogeny using; (i) treedater^17^, assuming a relaxed clock with the best root selected from 5 candidates selected from a root-to-tip analysis; (ii) LSD2^18^, with the root selected from candidates around the root identified by the root-to-tip analysis (using the R wrapper Rlsd2); and (iii) TreeTime^19^, assuming a relaxed clock (slack parameter 1.0, coupling parameter 0.0). The resulting trees were then pruned to include only the index cases and named partners, and patristic distances were calculated using the get_pairwise_distances function in the R library castor. Patristic distances were compared to nucleotide distances calculated using the tn93 program (<https://github.com/veg/tn93>).

1. **Named partners with HIV diagnoses following completion of contact tracing of index persons with IHI**

During Partner Services (PS), 19 contacts were named by an index person (n=18) that were HIV-negative (n=15) or HIV-status unknown (n=4 not located/refused PS) who subsequently tested HIV-positive following completion of contact tracing (>180 days after the IHI diagnosis). Overall, this implies over 8% incidence over the 2014-2018 period for individuals initially reported as HIV negative or status unknown (19/232).

| **Table S1. Characteristics of Persons with HIV Diagnosed in North Carolina, 2014-2018** | | | | |
| --- | --- | --- | --- | --- |
| **Characteristic** | **≥1 Sequence in Analysis** | | **NC Diagnoses 2014-2018** | |
|  | n | col% (CI) | n | col% (CI) |
| **Total** | **4138** | **63** | **6543** | **100** |
| **Race/ethnicity** |  |  |  |  |
| African American | 2650 | 64 (63,66) | 4114 | 63 (62,64) |
| White | 915 | 22 (21,23) | 1477 | 23 (22,24) |
| Latino/a | 381 | 9 (8-10) | 646 | 10 (9,11) |
| Other | 192 | 5 (4,5) | 306 | 5 (4,5) |
| **NC Field Services Unit Region at Diagnosis** |  |  |  |  |
| Asheville | 179 | 4 (4,5) | 391 | 6 (5,7) |
| Charlotte | 1047 | 25 (24,27) | 1695 | 26 (25,27) |
| Greensboro | 899 | 22 (20,23) | 1322 | 20 (19,21) |
| Raleigh | 922 | 22 (21,24) | 1331 | 20 (19,21) |
| Fayetteville | 328 | 8 (7,9) | 681 | 10 (10,11) |
| Winterville | 518 | 13 (12,14) | 780 | 12 (11,13) |
| Wilmington | 245 | 6 (5,7) | 343 | 5 (5,6) |
| **Age at HIV diagnosis** |  |  |  |  |
| 13-17 years old | 58 | 1 (1,2) | 87 | 1 (1,2) |
| 18-24 years old | 1088 | 26 (25,28) | 1681 | 26 (25,27) |
| 25-34 years old | 1328 | 32 (31,34) | 2102 | 32 (31,33) |
| 35-44 years old | 681 | 16 (15,18) | 1089 | 17 (16,18) |
| ≥45 years old | 983 | 24 (22,25) | 1584 | 24 (23,25) |
| **Gender** |  |  |  |  |
| Female | 773 | 19 (18,20) | 1292 | 20 (19,21) |
| Male | 3337 | 81 (79,82) | 5197 | 79 (78,80) |
| Transgender | 28 | 1 (<1,1) | 54 | 1 (<1,1) |
| **HIV Transmission Risk** |  |  |  |  |
| MSM | 2391 | 58 (56,59) | 3630 | 55 (54,57) |
| IDU | 101 | 2 (2,3) | 162 | 2 (2,3) |
| MSM+IDU | 109 | 3 (2,3) | 163 | 2 (2,3) |
| Heterosexual | 1531 | 37 (36,38) | 2568 | 39 (38,40) |
| Other/Unknown | 6 | <1 (<1,<1) | 20 | <1 (<1,<1) |
| **Year of HIV Diagnosis** |  |  |  |  |
| 2014 | 728 | 18 (16,19) | 1316 | 20 (19,21) |
| 2015 | 859 | 21 (20,22) | 1339 | 20 (19,21) |
| 2016 | 988 | 24 (23,25) | 1387 | 21 (20,22) |
| 2017 | 858 | 21 (20,22) | 1297 | 20 (19,21) |
| 2018 | 705 | 17 (16,18) | 1204 | 18 (17,19) |

| Table S2. Characteristics of members of clusters including ≥1 person diagnosed with incident HIV infection (IHI), 2014-2018 | | | | | | |
| --- | --- | --- | --- | --- | --- | --- |
|  | **<2014 (N=298)** | | **2014-2018 (N=1003)** | | **Total (N=1301)** | |
| **IHI 2014-2019,** n % (CI) |  |  |  |  |  |  |
| Not IHI | 298 | 100 (99, 100) | 770 | 77 (74, 79) | 1068 | 82 (80, 84) |
| IHI 2014-2018 | 0 | 0 (0, 1) | 233 | 23 (21, 26) | 233 | 18 (16, 20) |
| **Sex at Birth,** n % (CI) |  |  |  |  |  |  |
| Male | 289 | 97 (94, 99) | 936 | 93 (92, 95) | 1225 | 94 (93, 95) |
| Female | 9 | 3 (1, 6) | 67 | 7 (5, 8) | 76 | 6 (5, 7) |
| **Race/Ethnicity,** n % (CI) |  |  |  |  |  |  |
| Black | 226 | 76 (71, 81) | 669 | 67 (64, 70) | 895 | 69 (66, 71) |
| White | 41 | 14 (10, 18) | 217 | 22 (19, 24) | 258 | 20 (18, 22) |
| Latino | 12 | 4 (2, 7) | 73 | 7 (6, 9) | 85 | 7 (5, 8) |
| Other | 19 | 6 (4, 10) | 44 | 4 (3, 6) | 63 | 5 (4, 6) |
| **Age <30 at Diagnosis,** n % (CI) |  |  |  |  |  |  |
| Yes | 229 | 77 (72, 82) | 709 | 71 (68, 73) | 938 | 72 (70, 75) |
| No | 69 | 23 (18, 28) | 294 | 29 (27, 32) | 363 | 28 (25, 30) |
| **Age at first PRRT sequence,** median (IQR) | 27 | (23,33) | 26 | (23,32) | 26 | (23,32) |
| **MSM Risk Group,** n % (CI) |  |  |  |  |  |  |
| Yes | 235 | 79 (74, 83) | 799 | 80 (77, 82) | 1034 | 79 (77, 82) |
| No | 63 | 21 (17, 26) | 204 | 20 (18, 23) | 267 | 21 (18, 23) |
| **Viral link <1.5% to a IHI index,** n % (CI) | 163 | 55 (49, 60) | 810 | 81 (78, 83) | 973 | 75 (72, 77) |
| **Ever Linked IHI with Dx <90d IHI,** n % (CI) |  |  |  |  |  |  |
| Yes | 161 | 54 (48, 60) | 226 | 23 (20, 25) | 387 | 30 (27, 32) |
| No | 137 | 46 (40, 52) | 777 | 77 (75, 80) | 914 | 70 (68, 73) |
| **Number of Prior Links,** median (IQR) | 1 | (1, 1) | 1 | (1,2) | 1 | (1,1) |
| **Years from Diagnosis to IHI Diagnosis,** median (IQR) | -4 | (-7, -3) | -1 | (-2, -1) | -2 | (-3, -1) |
| **No viral suppression in 2018,** n % (CI) |  |  |  |  |  |  |
| Yes | 136 | 46 (40, 51) | 249 | 25 (22, 28) | 385 | 30 (27, 32) |
| No | 162) | 54 (49, 60) | 754 | 75 (72, 78) | 916 | 70 (68, 73) |
| **Field Services Unit region,** n % (CI) |  |  |  |  |  |  |
| Asheville | 14 | 5 (3, 8) | 40 | 4 (3, 5) | 54 | 4 (3, 5) |
| Charlotte | 69 | 23 (18, 28) | 232 | 23 (21, 26) | 301 | 23 (21, 26) |
| Greensboro | 43 | 14 (11, 19) | 229 | 23 (20, 26) | 272 | 21 (19, 23) |
| Raleigh | 107 | 36 (30, 42) | 282) | 28 (25, 31) | 389 | 30 (27, 32) |
| Fayetteville | 25 | 8 (6, 12) | 65 | 6 (5, 8) | 90 | 7 (6, 8) |
| Winterville | 34 | 11 (8, 16) | 108 | 11 (9, 13) | 142 | 11 (9, 13) |
| Wilmington | 6 | 2 (1, 4) | 47 | 5 (3, 6) | 53 | 4 (3, 5) |

| Table S3. Characteristics of persons with prior diagnoses (<2014) who are members of clusters that include an IHI index person clusters compared to those who are not members of clusters with IHI index persons | | | | | | |
| --- | --- | --- | --- | --- | --- | --- |
|  | **Not in cluster (N=9821)** | | **In Cluster (N=298)** | | **Total (N=10119)** | |
| **Race/Ethnicity,** n % (CI) |  |  |  |  |  |  |
| Black | 6997 | 71 (70, 72) | 226 | 76 (71, 81) | 7223 | 71 (70, 72) |
| White | 1896 | 19 (19, 20) | 41 | 14 (10, 18) | 1937 | 19 (18, 20) |
| Latino | 461 | 5 (4, 5) | 12 | 4 (2, 7) | 473 | 5 (4, 5) |
| Other | 467 | 5 (4, 5) | 19 | 6 (4, 10) | 486 | 5 (4, 5) |
| **Age <26 years at Diagnosis,** n % (CI) |  |  |  |  |  |  |
| Yes | 2941 | 30 (29, 31) | 187 | 63 (57, 68) | 3128 | 31 (30, 32) |
| No | 6880 | 70 (69, 71) | 111 | 37 (32, 43) | 6991 | 69 (68, 70) |
| **MSM Risk Group,** n % (CI) |  |  |  |  |  |  |
| Yes | 3720 | 38 (37, 39) | 235 | 79 (74, 83) | 3955 | 39 (38, 40) |
| No | 6101 | 62 (61, 63) | 63 | 21 (17, 26) | 6164 | 61 (60, 62) |
| **Mean % VL >1500,** median (IQR) | 25 | (1,68) | 35 | (6,81) | 26 | (1,69) |
| **No viral suppression in 2018,** n % (CI) |  |  |  |  |  |  |
| Yes | 3267 | 33 (32, 34) | 136 | 46 (40, 51) | 3403 | 34 (33, 35) |
| No | 6554 | 67 (66, 68) | 162 | 54 (49, 60) | 6716 | 66 (65, 67) |

**Figure S1.** Sinaplots showing the pairwise genetic distance using the TN-93 model among n=14,524 persons. Boxplot shows the median and IQR for each group.

| 1. 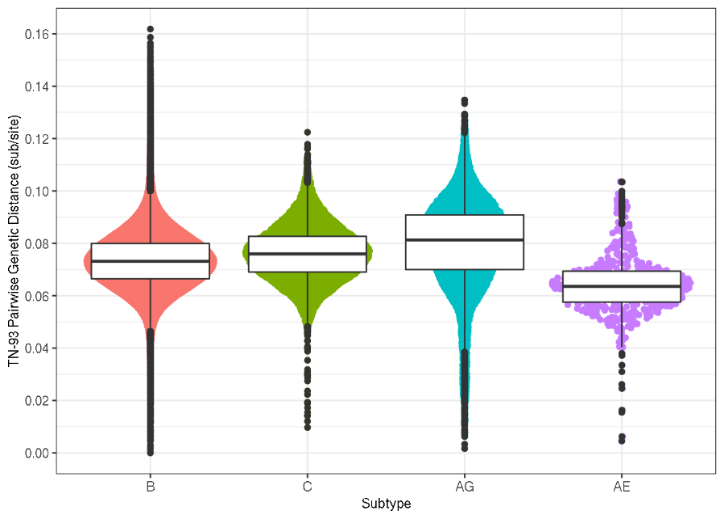Pairwise Genetic Distances among all Persons, Stratified by four major subtypes | 1. Minimum Pairwise Genetic Distance per Person, Stratified by Subtype   **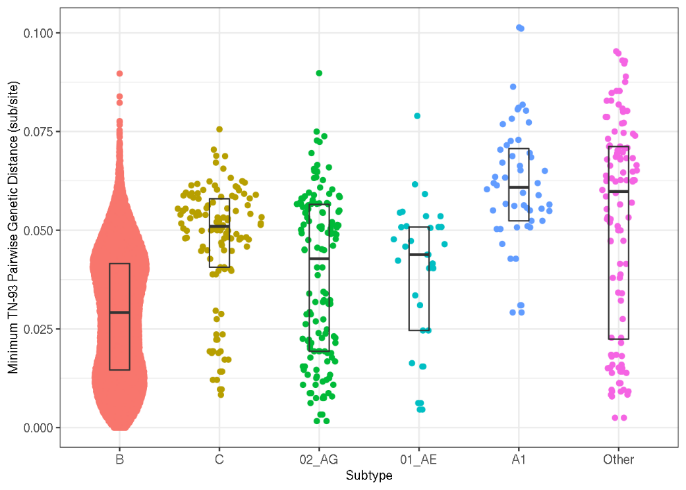** |
| --- | --- |
| 1. Minimum Pairwise Genetic Distance per Person, Stratified by Membership in a Cluster with an Incident HIV Infection (IHI)   **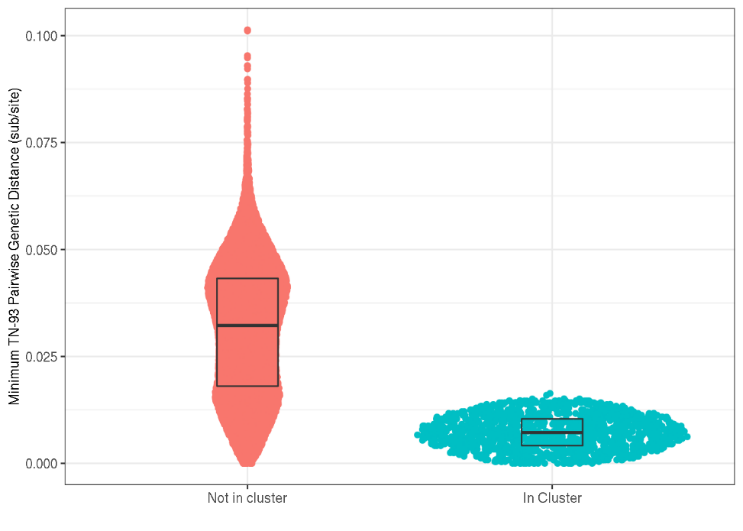** |  |

**Figure S2.** Distribution of minimum TN-93 pairwise genetic distances for Recent Diagnoses (2014-2018) [A-B] and Index Persons with Incident HIV infection (IHI) [C-D].

| 1. Distribution of Minimum Pairwise Genetic Distance for Recent Diagnoses (2014-2018), n=4,405 persons. Vertical blue line indicates median.   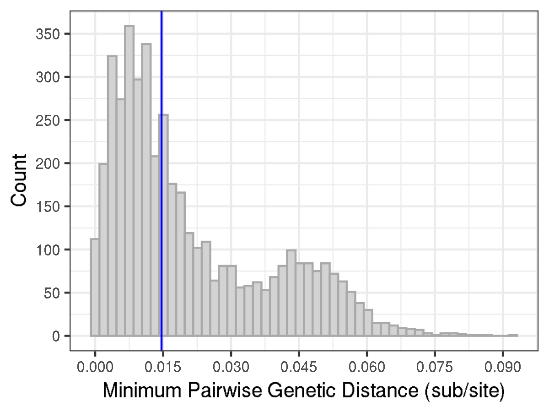 | 1. Density distribution finite mixture model with the Expectation-Maximum (E-M) algorithm for Recent Diagnoses. Vertical blue line indicates estimated cutoff.   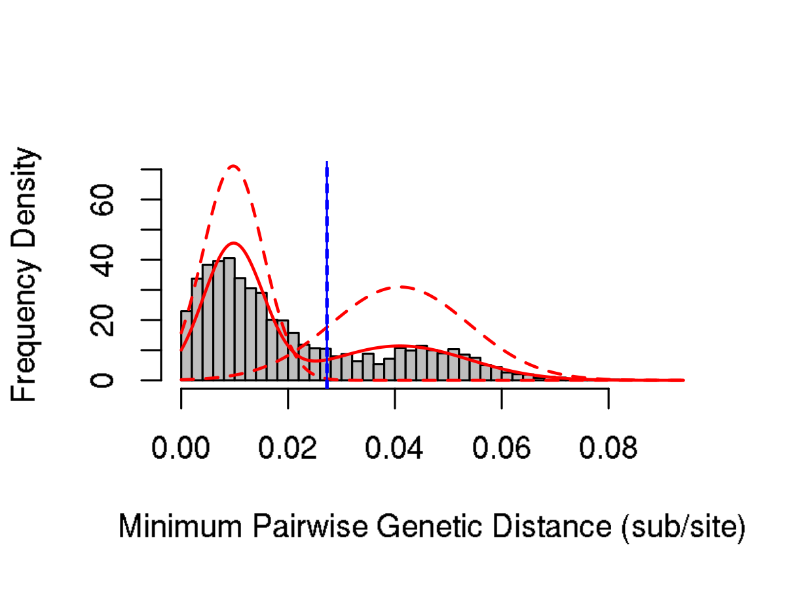 |
| --- | --- |
| 1. Distribution of Minimum Pairwise Genetic Distance for Index Persons, n=323 persons. Vertical blue line indicates median.   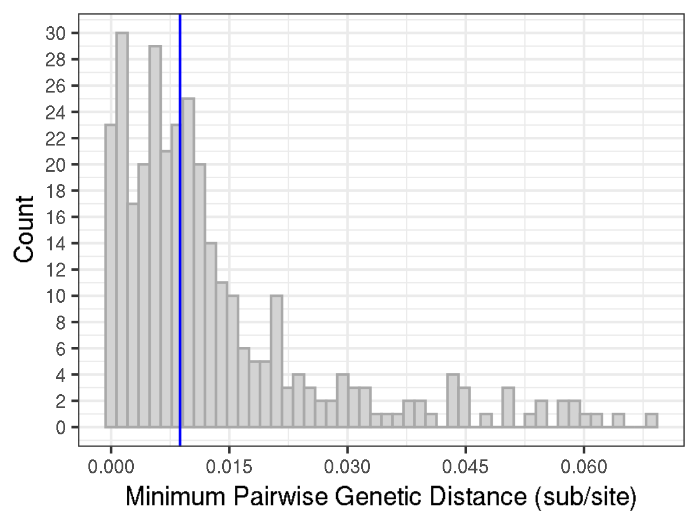 | 1. Density finite mixture model with the Expectation-Maximum (E-M) algorithm for Index Persons. Vertical blue line indicates estimated cutoff.   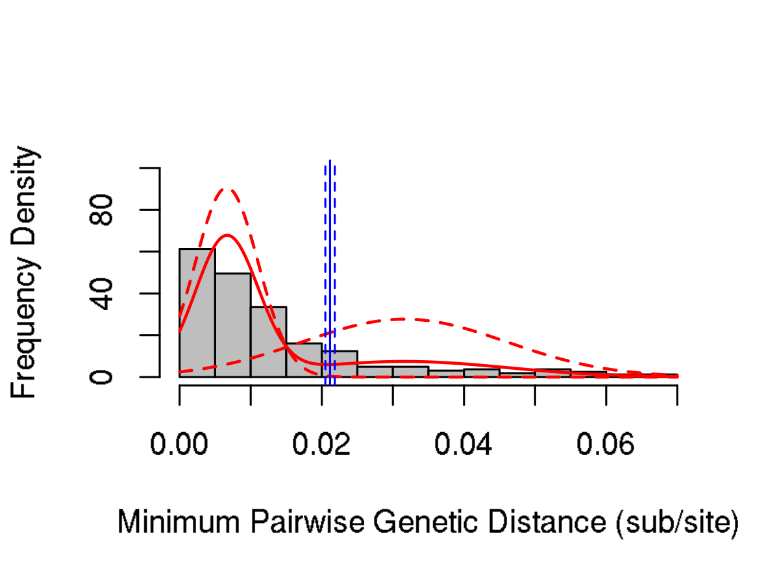 |

**Figure S3.** Distribution of HIV-positive persons with genetic links <1.5% to an incident HIV infection (IHI)

**
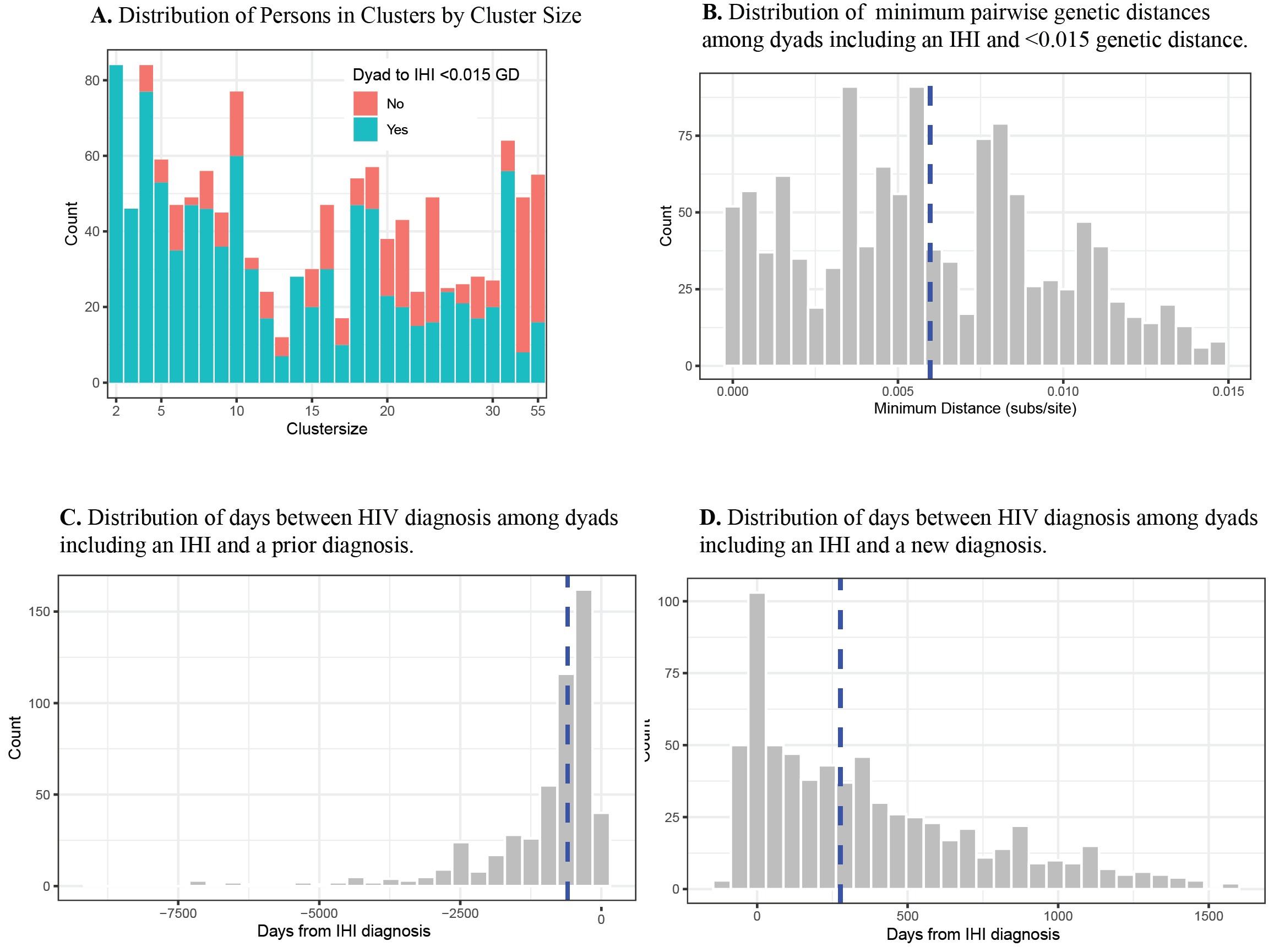
**

**Figure S4.** Named Partner Contact Network. Genetic edges shown are dyads <0.015 pairwise genetic distance.


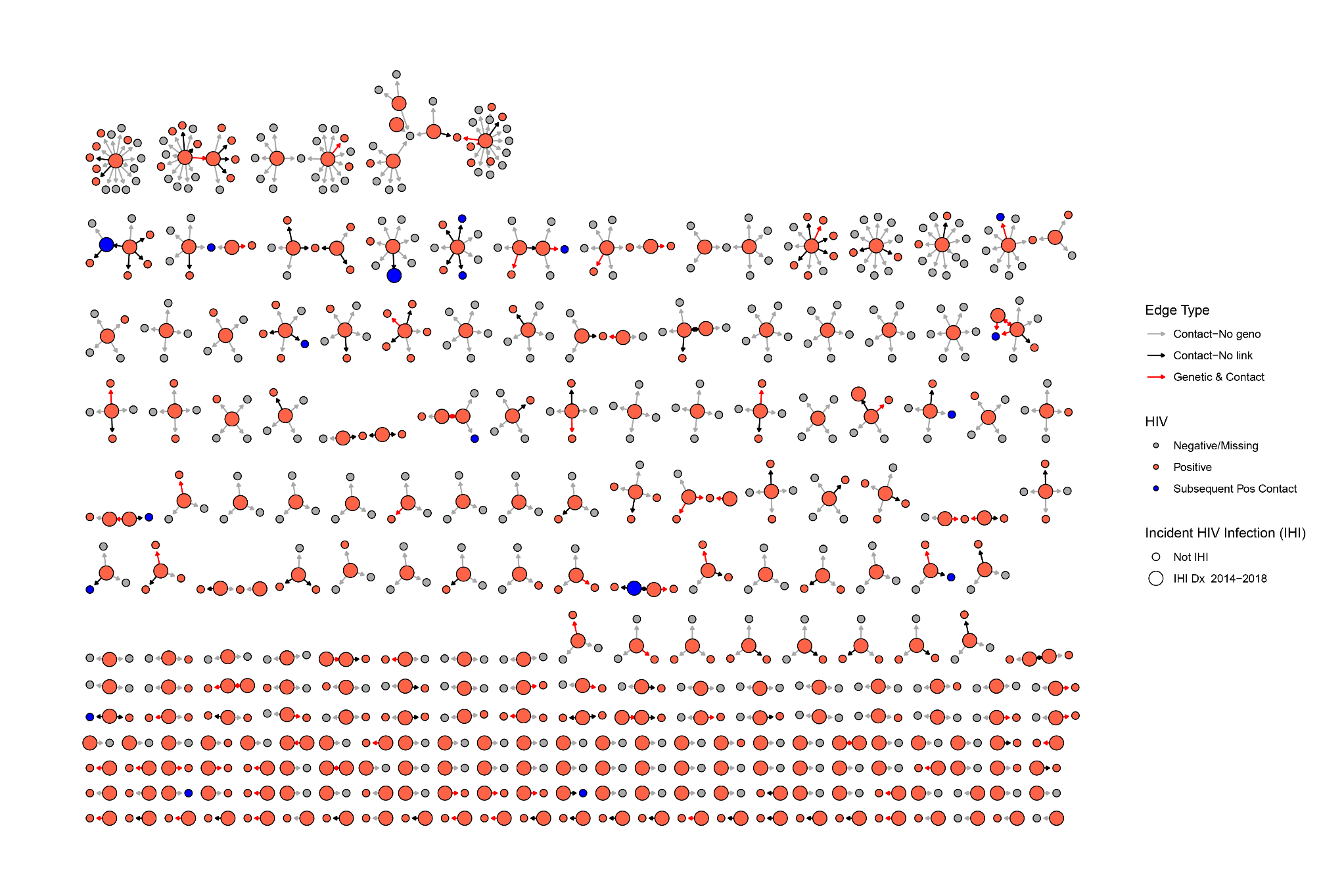


**Figure S5.** Distribution of Pairwise Genetic Distances (sub/site) for Index Persons with incident HIV infection (IHI) ­­ and Named Partners

| 1. Distribution of Pairwise Genetic Distances (sub/site) for Index Persons and Named Partners. Colors differentiate between distances at 0.030 cutoff.   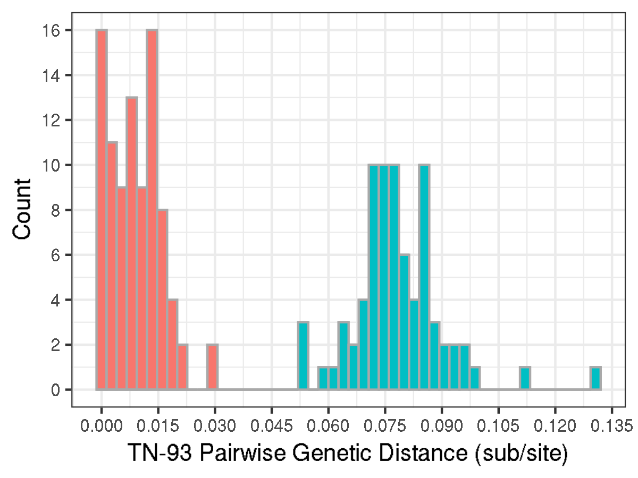 | 1. Days between Index and Named Partner Sequence Collection Dates. Colors differentiate between distances at 0.030 cutoff.   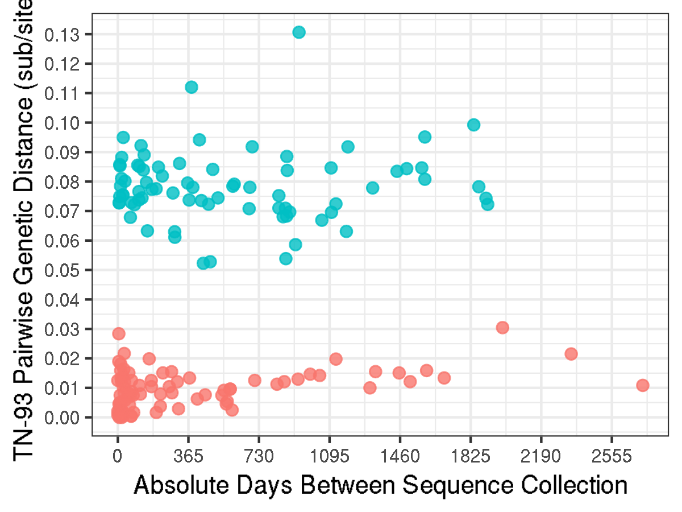 |
| --- | --- |
| 1. Days from Index Diagnosis and Partner Sequence Collection. Colors differentiate between distances at 0.030 cutoff.     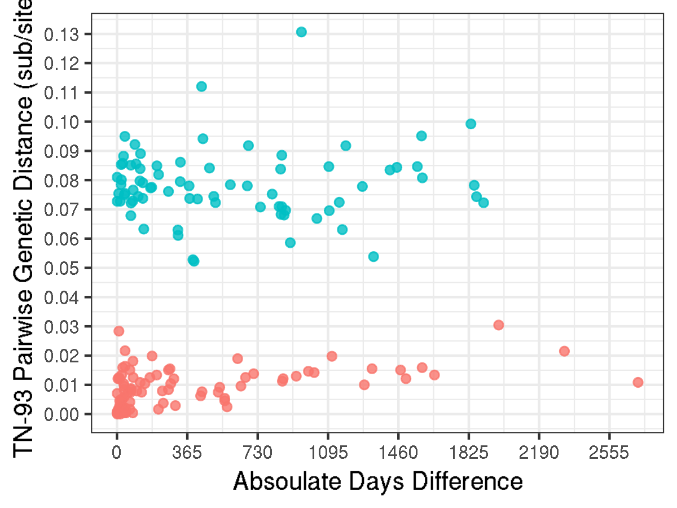 | 1. Days from Index Diagnosis and Partner Sequence Collection for Potential Transmission Pairs (Mode 1 in Fig. S3A). Shapes indicate whether dyad included a new (circle) versus prior diagnosis (triangle).   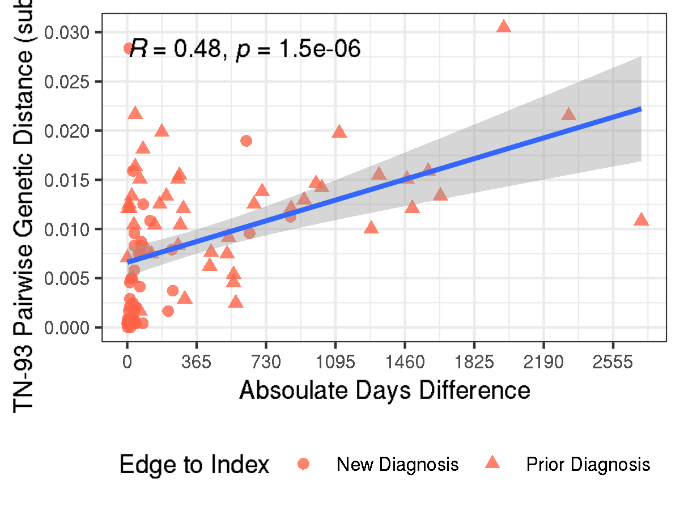 |

**
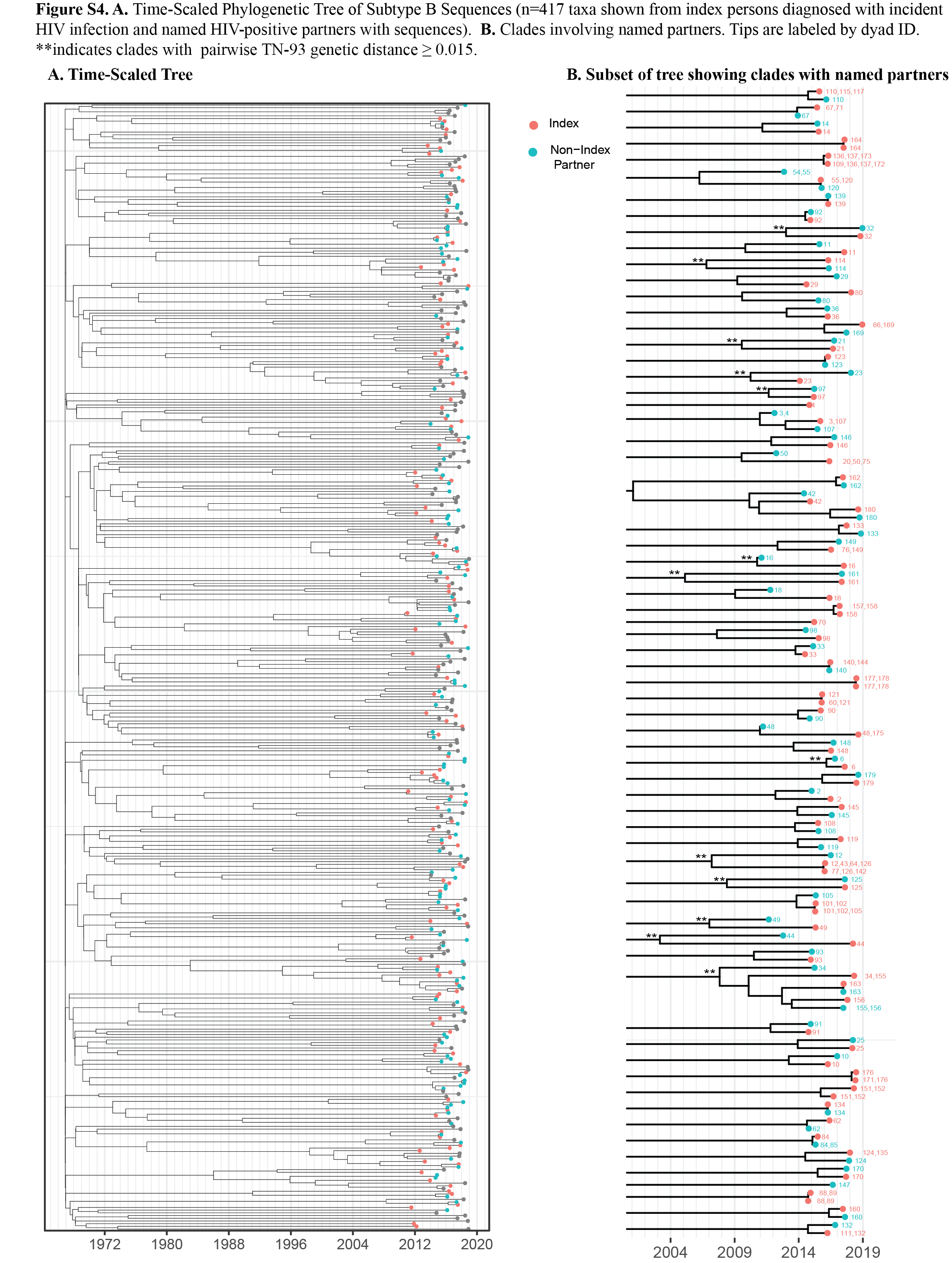
Figure S6. A.** Time-Scaled Phylogenetic tree of subtype B sequences (n=417 taxa shown from index persons diagnosed with incident HIV infection and named HIV-positive partners). B. Clades involving named partners. Tips are labeled by id number referencing named dyads indivdual belongs to. **indicates clades wth pairwise TN-93 distance ≥0.015. Tree shown generated in treedater.^17^

**Figure S7.** Network of Genetic Clusters and Named Partner Contacts. Genetic edges shown are dyads <0.015 pairwise genetic distance.


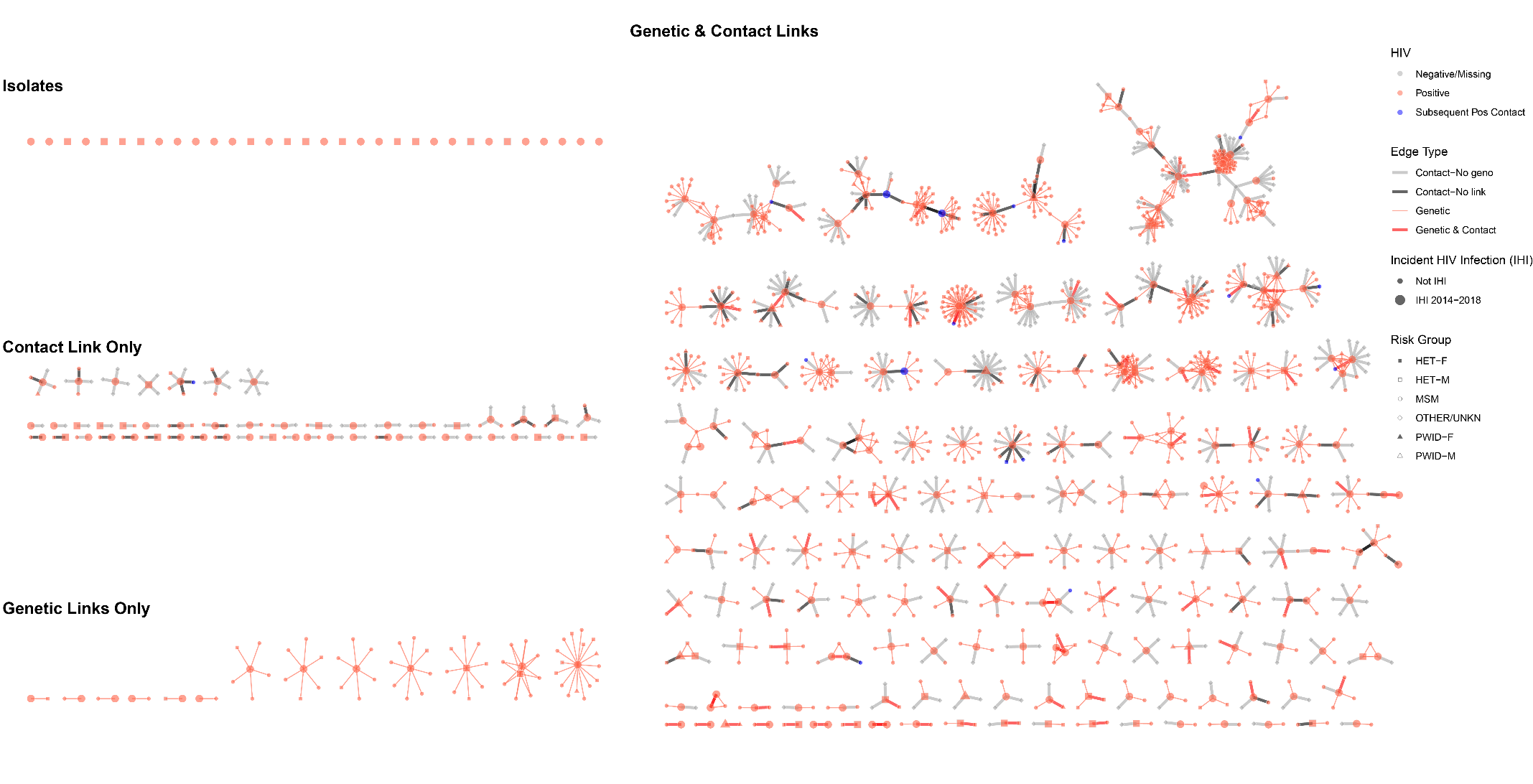


**REFERENCES**

1 2018 North Carolina' ' HIV Surveillance Report. 2019; published online Aug. https://epi.dph.ncdhhs.gov/cd/stds/annualrpts.html (accessed July 19, 2020).

2 Tamura K, Nei M. Estimation of the number of nucleotide substitutions in the control region of mitochondrial DNA in humans and chimpanzees. *Mol Biol Evol* 1993; **10**: 512–526.

3 Pineda-Peña A-C, Faria NR, Imbrechts S, *et al.* Automated subtyping of HIV-1 genetic sequences for clinical and surveillance purposes: performance evaluation of the new REGA version 3 and seven other tools. *Infect Genet Evol* 2013; **19**: 337–348.

4 Struck D, Lawyer G, Ternes A-M, Schmit J-C, Bercoff DP. COMET: adaptive context-based modeling for ultrafast HIV-1 subtype identification. *Nucleic Acids Res* 2014; **42**: e144.

5 Bennett DE, Camacho RJ, Otelea D, *et al.* Drug resistance mutations for surveillance of transmitted HIV-1 drug-resistance: 2009 update. *PLoS One* 2009; **4**: e4724.

6 Novitsky V, Moyo S, Essex M. Phylogenetic inference of HIV transmission clusters. *Infectious Diseases and Translational Medicine* 2017; **3**: 51–59.

7 Oster AM, France AM, Panneer N, *et al.* Identifying Clusters of Recent and Rapid HIV Transmission Through Analysis of Molecular Surveillance Data. *J Acquir Immune Defic Syndr* 2018; **79**: 543–550.

8 Poon AFY. Impacts and shortcomings of genetic clustering methods for infectious disease outbreaks. *Virus Evol* 2016; **2**: vew031.

9 Junqueira DM, Sibisi Z, Wilkinson E, de Oliveira T. Factors influencing HIV-1 phylogenetic clustering. *Curr Opin HIV AIDS* 2019; **14**: 161–172.

10 Hightower GK, May SJ, Pérez-Santiago J, *et al.* HIV-1 clade B pol evolution following primary infection. *PLoS One* 2013; **8**: e68188.

11 Ratmann O, Grabowski MK, Hall M, *et al.* Inferring HIV-1 transmission networks and sources of epidemic spread in Africa with deep-sequence phylogenetic analysis. *Nat Commun* 2019; **10**: 1411.

12 Wertheim JO, Kosakovsky Pond SL, Forgione LA, *et al.* Social and Genetic Networks of HIV-1 Transmission in New York City. *PLoS Pathog* 2017; **13**: e1006000.

13 Trang NV, Choisy M, Nakagomi T, *et al.* Determination of cut-off cycle threshold values in routine RT-PCR assays to assist differential diagnosis of norovirus in children hospitalized for acute gastroenteritis. *Epidemiol Infect* 2015; **143**: 3292–3299.

14 Price MN, Dehal PS, Arkin AP. FastTree 2 — approximately maximum-likelihood trees for large alignments. *PLoS One* 2010; **5**: e9490.

15 Minh BQ, Schmidt HA, Chernomor O, *et al.* IQ-TREE 2: New Models and Efficient Methods for Phylogenetic Inference in the Genomic Era. *Mol Biol Evol* 2020; **37**: 1530–1534.

16 Paradis E, Schliep K. ape 5.0: an environment for modern phylogenetics and evolutionary analyses in R. *Bioinformatics* 2019; **35**: 526–528.

17 Volz EM, Frost SDW. Scalable relaxed clock phylogenetic dating. *Virus Evol* 2017; **3**. DOI:10.1093/ve/vex025.

18 To T-H, Jung M, Lycett S, Gascuel O. Fast Dating Using Least-Squares Criteria and Algorithms. *Syst Biol* 2016; **65**: 82–97.

19 Sagulenko P, Puller V, Neher RA. TreeTime: Maximum-likelihood phylodynamic analysis. *Virus Evol* 2018; **4**: vex042.

STROBE Statement—checklist of items that should be included in reports of observational studies.

|  | Item No | Recommendation | Checklist |
| --- | --- | --- | --- |
| **Title and abstract** | 1 | (*a*) Indicate the study’s design with a commonly used term in the title or the abstract | √ |
|  |  | (*b*) Provide in the abstract an informative and balanced summary of what was done and what was found | √ |
| Introduction | | |  |
| Background/rationale | 2 | Explain the scientific background and rationale for the investigation being reported | √ |
| Objectives | 3 | State specific objectives, including any prespecified hypotheses |  |
| Methods | | | √ |
| Study design | 4 | Present key elements of study design early in the paper |  |
| Setting | 5 | Describe the setting, locations, and relevant dates, including periods of recruitment, exposure, follow-up, and data collection | √ |
| Participants | 6 | (*a*) *Cross-sectional study*—Give the eligibility criteria, and the sources and methods of selection of participants | √ |
|  |  | (*b*) *Cohort study*—For matched studies, give matching criteria and number of exposed and unexposed *Case-control study*—For matched studies, give matching criteria and the number of controls per case | n/a |
| Variables | 7 | Clearly define all outcomes, exposures, predictors, potential confounders, and effect modifiers. Give diagnostic criteria, if applicable | √ |
| Data sources/ measurement | 8* | For each variable of interest, give sources of data and details of methods of assessment (measurement). Describe comparability of assessment methods if there is more than one group | √ |
| Bias | 9 | Describe any efforts to address potential sources of bias | √ |
| Study size | 10 | Explain how the study size was arrived at | √ |
| Quantitative variables | 11 | Explain how quantitative variables were handled in the analyses. If applicable, describe which groupings were chosen and why | √ |
| Statistical methods | 12 | (*a*) Describe all statistical methods, including those used to control for confounding | √ |
|  |  | (*b*) Describe any methods used to examine subgroups and interactions | √ |
|  |  | (*c*) Explain how missing data were addressed | √ |
|  |  | (*d*) *Cross-sectional study*—If applicable, describe analytical methods taking account of sampling strategy | √ |
|  |  | (*e*) Describe any sensitivity analyses | n/a |
| Results |  |  |  |
| Participants | 13* | (a) Report numbers of individuals at each stage of study—eg numbers potentially eligible, examined for eligibility, confirmed eligible, included in the study, completing follow-up, and analysed | √ |
|  |  | (b) Give reasons for non-participation at each stage | √ |
|  |  | (c) Consider use of a flow diagram | √ |
| Descriptive data | 14* | (a) Give characteristics of study participants (eg demographic, clinical, social) and information on exposures and potential confounders | √ |
|  |  | (b) Indicate number of participants with missing data for each variable of interest | √ |
|  |  | (c) *Cohort study*—Summarise follow-up time (eg, average and total amount) | n/a |
| Outcome data | 15* | *Cohort study*—Report numbers of outcome events or summary measures over time | n/a |
|  |  | *Case-control study—*Report numbers in each exposure category, or summary measures of exposure | n/a |
|  |  | *Cross-sectional study—*Report numbers of outcome events or summary measures | √ |
| Main results | 16 | (*a*) Give unadjusted estimates and, if applicable, confounder-adjusted estimates and their precision (eg, 95% confidence interval). Make clear which confounders were adjusted for and why they were included | √ |
|  |  | (*b*) Report category boundaries when continuous variables were categorized | √ |
|  |  | (*c*) If relevant, consider translating estimates of relative risk into absolute risk for a meaningful time period | n/a |
| Other analyses | 17 | Report other analyses done—eg analyses of subgroups and interactions, and sensitivity analyses | √ |
| Discussion |  |  |  |
| Key results | 18 | Summarise key results with reference to study objectives | √ |
| Limitations | 19 | Discuss limitations of the study, taking into account sources of potential bias or imprecision. Discuss both direction and magnitude of any potential bias | √ |
| Interpretation | 20 | Give a cautious overall interpretation of results considering objectives, limitations, multiplicity of analyses, results from similar studies, and other relevant evidence | √ |
| Generalisability | 21 | Discuss the generalisability (external validity) of the study results | √ |
| Other information |  |  |  |
| Funding | 22 | Give the source of funding and the role of the funders for the present study and, if applicable, for the original study on which the present article is based | √ |
